# IgG3 collaborates with IgG1 and IgA to recruit effector function in RV144 vaccinees

**DOI:** 10.1172/jci.insight.140925

**Published:** 2020-11-05

**Authors:** Stephanie Fischinger, Sepideh Dolatshahi, Madeleine F. Jennewein, Supachai Rerks-Ngarm, Punnee Pitisuttithum, Sorachai Nitayaphan, Nelson Michael, Sandhya Vasan, Margaret E. Ackerman, Hendrik Streeck, Galit Alter

**Affiliations:** 1 Ragon Institute of MGH, MIT and Harvard, Cambridge, Massachusetts, USA.; 2 PhD Program of Virology and Immunology, University of Duisburg-Essen, Essen, Germany.; 3 University of Virginia, Charlottesville, Virginia, USA.; 4 Thai Ministry of Public Health, Nonthaburi, Thailand.; 5 Mahidol University, Bangkok, Thailand.; 6 Royal Thai Army Component, AFRIMS, Bangkok, Thailand.; 7 U.S. Military HIV Research Program, Walter Reed Army Institute of Research, Silver Spring, Maryland, USA.; 8 Henry M. Jackson Foundation for the Advancement of Military Medicine, Bethesda, Maryland, USA.; 9 Thayer School of Engineering, Dartmouth College, Hanover, New Hampshire, USA.; 10 Institute of Virology, Universitätsklinikum Bonn, Bonn, Germany.

**Keywords:** AIDS/HIV, Vaccines, AIDS vaccine, Adaptive immunity, Cellular immune response

## Abstract

While the RV144 HIV vaccine trial led to moderately reduced risk of HIV acquisition, emerging data from the HVTN702 trial point to the critical need to reexamine RV144-based correlates of reduced risk of protection. While in RV144, the induction of V2-binding, non-IgA, IgG3 antibody responses with nonneutralizing functions were linked to reduced risk of infection, the interactions between these signatures remain unclear. Thus, here we comprehensively profile the humoral immune response in 300 RV144 vaccinees to decipher the relationships between humoral biomarkers of protection. We found that vaccine-specific IgG1, IgG3, and IgA were highly correlated. However, ratios of IgG1:IgG3:IgA provided insights into subclass/isotype polyclonal functional regulation. For instance, in the absence of high IgG1 levels, IgG3 antibodies exhibited limited functional activity, pointing to IgG3 as a critical contributor, but not sole driver, of effective antiviral humoral immunity. Higher IgA levels were linked to enhanced antibody effector function, including neutrophil phagocytosis (ADNP), complement deposition (ADCD), and antibody-dependent NK degranulation (CD107a), some of which were increased in infected vaccinees in a case/control data set, suggesting that IgA-driven functions compromised immunity. These data highlight the interplay between IgG1, IgG3, and IgA, pointing to the need to profile the relationships between subclass/isotype selection.

## Abstract 2

The induction of V2-binding, non-IgA, IgG3 antibody responses with non-neutralizing functions were linked to reduced risk of infection in RV144 vaccinees.

## Introduction

Despite the extraordinary impact of globally expanded treatment and prevention efforts, aimed at controlling HIV disease and spread, access to antiretroviral (ARV) drugs remains difficult in many parts of the globe — for some populations, enabling the virus to continue to spread. Thus, the ultimate global control of HIV will require the development of a safe and effective HIV vaccine (1, 2). Despite the promising results from the RV144 HIV vaccination trial (3), the disappointing failure of the South African repeat trial, HVTN702, has raised questions related to the original RV144 correlates of immunity.

In the RV144 trial, a 60% and 31.2% reduction of infection was observed at 12 months and 2 years after vaccination, respectively, among vaccinees (3, 4), suggesting early but waning vaccine-induced immunity. While neither neutralizing antibodies nor cytotoxic T cell responses were observed, reduced risk of infection was associated with vaccine-induced antibodies targeting the variable loop 2 region (V1V2), which mediated antibody-dependent cellular cytotoxicity (ADCC) (5). Conversely, enhanced risk of infection was noted in subjects with high levels of vaccine-specific IgA responses (6), thought to block IgG activity (7). However, follow-up analyses pointed to an additional association between vaccine-induced IgG3 levels and reduced risk of infection (8), which exhibited broad antibody effector function (9). However, despite the univariate importance of each of these correlates, polyclonal selection of antibody subclass/isotype is highly heterogeneous and must be probed collectively to fully define the immune landscape and correlate interactions.

Thus, in this study, we aimed to comprehensively and deeply characterize the functional role of the coevolving vaccine-specific IgG1, IgG3, and IgA response among a large group of RV144 vaccinees. We employed an unbiased, high-throughput systems serology approach (10) that analyzes both functional and biophysical profiles of polyclonal antibodies. Both complete and IgA-depleted plasma were tested to gain a deeper appreciation for the impact of antibody subpopulations in driving innate immune effector function. We found critical and unexpected interactions and functional dependencies across IgG subclasses, in such a way that no subclass alone was able to drive full polyfunctionality. Moreover, IgA1 was also critically involved in driving antibody effector functions — including neutrophil phagocytosis, complement activation, and NK cell degranulation — but effectively inhibited NK cell cytokine secretion. These data highlight the unique interactions between immune biomarkers associated with different risk of HIV infection, pointing to the critical importance of profiling interactions between correlates of immunity to fully explore potential mechanistic hypotheses underlying protection against HIV.

## Results

### RV144 vaccinees exhibit variable antibody isotype profiles.

**Figure 1:**
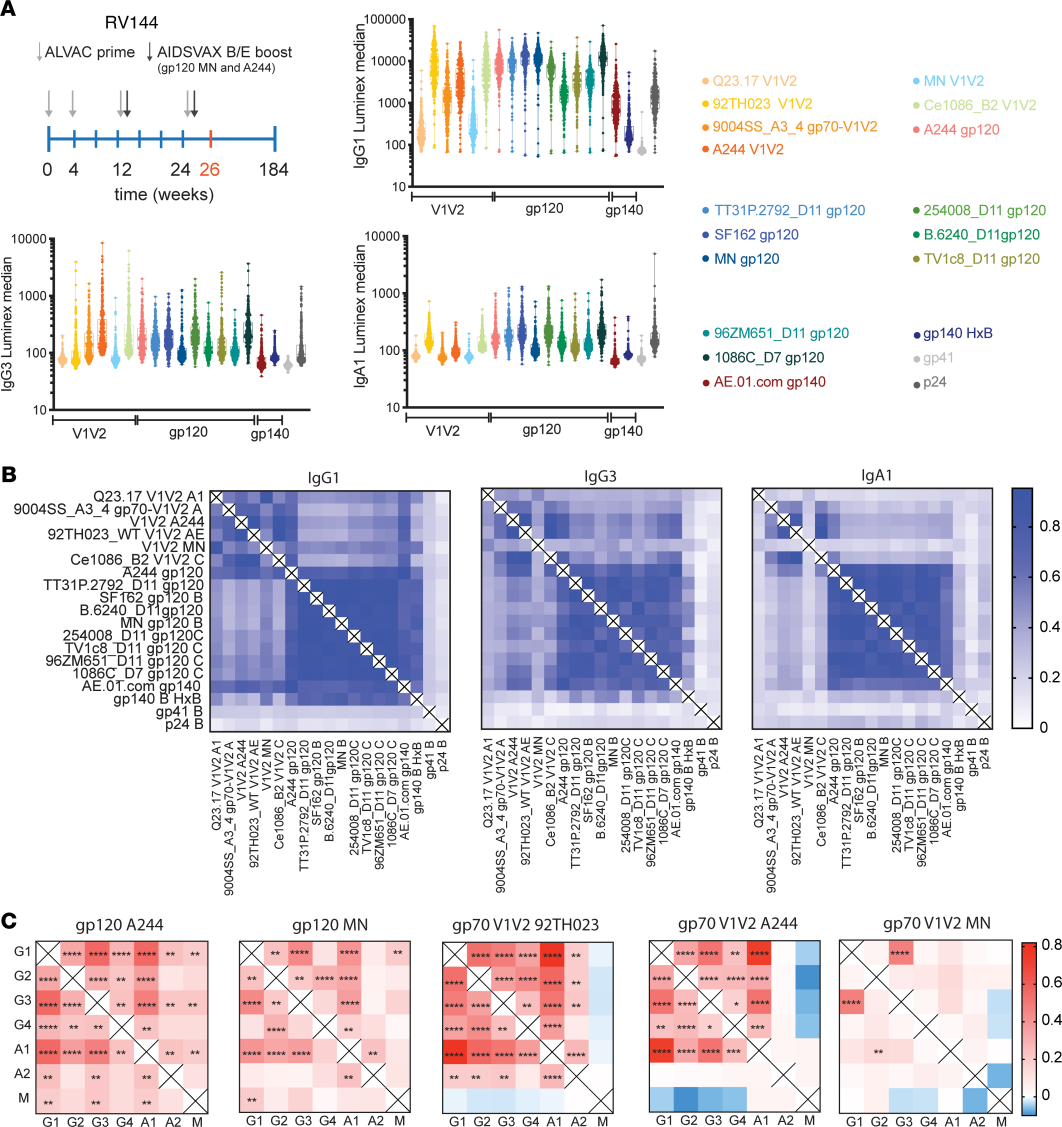
Overall profile of antibody responses in RV144 vaccinees. (**A**) A schematic of the vaccination schedule for RV144 is shown. Light gray arrows indicate the time point of ALVAC prime injection; dark gray arrows the administration of the AIDSVAX B/E boost. The samples analyzed in this study are from week 26 time point, indicated in red. The whisker plots depict the Luminex median fluorescence (MFI) as a relative measurement of antibody titer across 300 RV144 vaccinees and across various antigens from different clades and specificities. Antigens are grouped along the *x* axis by specificity, including Clade AE responses (red/orange), Clade B responses (blue), Clade C responses (green), and gp41/p24 responses (gray). IgG1, IgG3, and IgA antibody responses against different antigens are plotted; each dot represents the average of a duplicate run per sample. Minimum to maximum is shown in the box. (**B**) The correlation matrix depicts the pairwise combination of relative antibody levels for different antigens for IgG1, IgG3, and IgA1, assessed by Spearman’s correlation. Dark blue represents a strong correlation, whereas white indicates no correlation. (**C**) The correlation matrix shows the pairwise relationships of subclasses and isotypes within antigens using a Spearman’s correlation with Bonferroni’s correction for multiple comparisons. Red represents a positive correlation, and blue a negative relationship. Adjusted *P* value; *P < 0.05, **P < 0.01, ****P < 0.0001.

Previous immune correlate analysis of RV144 pointed to vaccine-induced differences in subclass and isotype distributions among vaccinees (11, 12). High levels of V1V2-specific IgG1 and IgG3 were classified as correlates of reduced risk of infection, whereas vaccinees with elevated IgA levels were found to be at higher risk of HIV acquisition (8, 13). However, the relationship between these univariate associations remain unclear, as well as how differing levels of antibody subclass/isotype selection influences antibody effector function. Given the complexity of subclass and isotype distributions across polyclonal sera, we therefore began probing the relationships between antibody subclass/isotype and functional responses in 300 RV144 vaccinees. Specifically, we assessed the overall level of vaccine-induced IgG1, IgG3, and IgA responses to a number of HIV envelope antigens, as well as to V1V2 scaffolds (13, 14), focusing on gp120 MN, one of the boost vaccine antigens for the characterization of functional responses. While antibody subclasses/isotype responses were detected in all vaccinees across all tested antigens, significant heterogeneity was observed across vaccine responses (Figure 1A). RV144 vaccinees elicited antibodies not only against the antigens directly included in the regimen (gp120 A244 and MN), but also across HIV clades (Clade C 254008, TV1c8) and strains (SF162, TT3P.2792). Importantly, while only a very small segment of gp41 was included in the vaccine (15), gp41 responses were still detectable in a few individuals. Antibody responses were also detected against gp120, gp140, and gp70 V1V2, as shown previously (16). However, cross-clade recognition varied across the subclasses/isotypes. For example, high levels of 92TH023 V1V2–directed IgG1 was observed in contrast to lower responses against the boosting antigens A244 and MN. IgG3 showed a distinct pattern, with a dominant response to the boosting antigens and a lesser response to the prime. For example, V1V2-specific IgG3 responses were skewed toward the V1V2 A244 antigen. In contrast, the IgA1 response showed a similar profile to IgG1 immunity, skewed toward the prime, but were generally shifted toward gp120 Clade B antigens. These data suggest that viral vector priming and protein antigen boosting may have differentially affected the immunodominance pattern of antigens recognized by the different subclasses/isotypes, highlighting the induction of potentially more recently selected V1V2-specific IgG3 responses to the boosting antigens, whereas V1V2-specific IgAs and IgG1s were induced preferentially to priming antigens. These data suggest that boosting may have skewed the subclasses/isotype response and that subjects able to respond to the boost-driven shift may have benefited preferentially from vaccination.

To gain a deeper appreciation for the polyclonal vaccine response profiles across the vaccinees, relationships across antibody responses were investigated to further understand the dynamics across antigens and antibody subclass/isotype selection (Figure 1B). Strong correlations were observed across all IgG1 specificities, demonstrating the broad recognition profiles of RV144-induced IgG1 responses, the dominant antibody subclass. Particularly, high correlations were observed within all V1V2 antigens and separately, within the gp120- and gp140-specific response, pointing to 2 different vaccine response profiles: a group that preferentially induced immunity to V1V2 and a second group that preferentially induced responses to the remaining vaccine antigens. However, this bimodal distribution profile was not observed among the IgG3 and IgA responses. Specifically, the response to the V1V2 region was less synchronized across both IgG3 and IgA responses, suggesting that IgG3- and IgA V2-specific immunity was induced in a more stochastic manner across antigens and across vaccinees. Moreover, while some level of coordination was observed between V1V2 and gp120/140 for IgG3 responses, coordination was solely observed between gp120 responses, and very low correlations were observed between gp140 antigens for the IgA response. These data argue for a unique and unexpected differential coordination of IgG1, IgG3, and IgA responses, in a distinct manner from IgG1, across antigens.

To therefore gain granular insights into the coordination of isotypes/subclasses, we next examined the relationships of isotype/subclass selection within antigens (Figure 1C). Differing patterns were observed across antigens and scaffolds. Gp120 antigens from both Clade AE and B were associated with coordinated responses, where A244 responses showed the greatest degree of correlation across nearly all isotype and subclasses. Conversely, MN-specific immune responses showed greater levels of variation. Greater heterogeneity in relationships was observed across V1V2 scaffold–specific responses, with remarkably strong coordination to the priming antigen 92TH023, moderate correlations to the A244 scaffold, and again more variable relationships to the MN V1V2 scaffold. Positive relationships were observed across most class-switched antibody subclasses/isotypes, that were largely anticorrelated to IgM responses for V1V2 scaffolds, but not gp120 antigens. These data strongly argue that a fully switched, mature humoral immune response was driven to the highly immunodominant V1V2 across all vaccinees, with MN-specific immunity marking the greatest divergence across vaccine-induced humoral immune responses and potentially marking the most heterogeneous (Figure 1A) and diverging (Figure 1, B and C) response to vaccination.

### IgG1, IgG3, and IgA are drivers of heterogeneous functional profiles in RV144 vaccinees.

**Figure 2:**
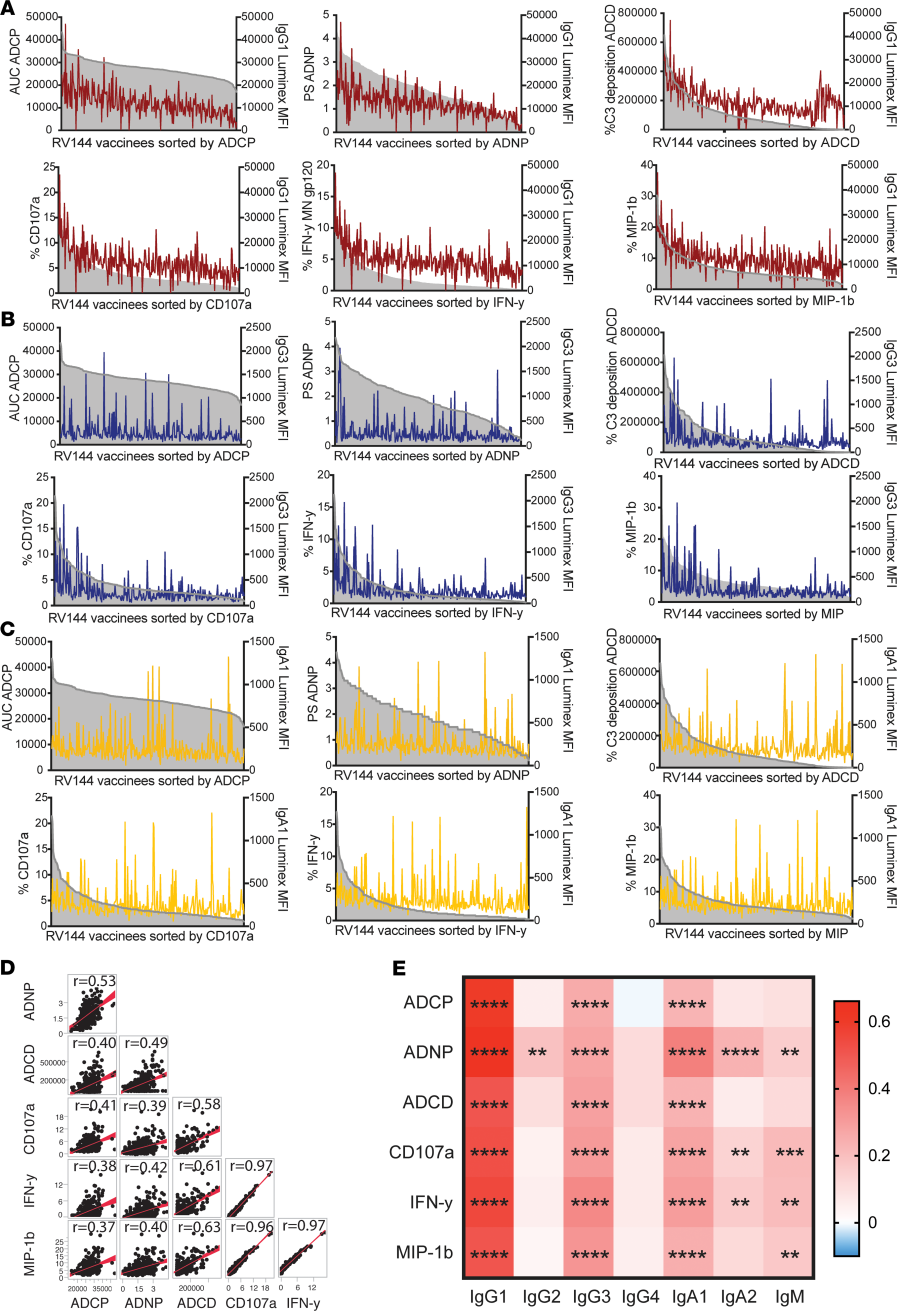
IgG1, IgG3, and IgA are correlated with the induction of effector functions. (**A**–**C**) Three hundred RV144 vaccinees were sorted by their ADCP, ADNP, ADCD, and NK cell (degranulation: %CD107a, %IFN-γ, and %MIP-1β) functional responses against gp120 MN, the vaccine boost antigen depicted in the shaded area of the graph. IgG1 (red) (**A**), IgG3 (**B**), and IgA1 (**C**) levels are depicted in the line to the same antigen. (**D**) The correlation scatter plot represents the relationship between all Fc-effector functions across all vaccinees. *R* values (Spearman’s correlation) are shown, and best fit line is indicated in red. (**E**) The heatmap shows a pairwise Spearman’s correlation matrix, Bonferroni corrected for multiple comparisons between effector functions and antibody titers against the gp120 MN antigen. Red represents a positive correlation, whereas blue indicates a negative correlation. Adjusted *P* value; ***P < 0.01, ****P < 0.0001).

Given the variation in subclass/isotype regulation across vaccinees, we next aimed to probe the functional consequences of these differences against the boosting antigen gp120 MN. The boost antigen gp120 MN was chosen for functional profiling, since previous studies have shown a high correlation between the 2 boost antigens for antibody-dependent cellular phagocytosis (ADCP) (9), which we confirmed in preliminary analysis (Supplemental Figure 1; supplemental material available online with this article; https://doi.org/10.1172/jci.insight.140925DS1). Thus, we next profiled the ability of vaccine-induced antibodies to drive ADCP, antibody-dependent complement deposition (ADCD), and antibody-dependent neutrophil phagocytosis (ADNP), as well as the ability to drive antibody-dependent NK cell activation (degranulation [CD107a],IFN-γ, and macrophage inflammatory protein-β [MIP-1β] secretion) against the boosting antigen gp120 MN antigen (Figure 2), which showed the greatest heterogeneity in subclass/isotype profiles (Figure 1). For each function, RV144 vaccinees were ordered by their level of functionality, with respect to their IgG1 and IgG3 titers for the same antigen. Substantial heterogeneity existed in antibody functional responses across the vaccinees (Figure 2, A–C). IgG1 levels tracked with all effector functions (i.e., individuals with high IgG1 levels also had higher functional responses). Conversely, IgG3 levels exhibited more erratic relationships with ADCP, ADNP, and ADCD but were enriched among individuals with the highest NK cell functions (Figure 2B). For IgA1, a weaker relation between IgA1 levels and functions can be seen, indicating that the contribution of IgA1 to functions is weaker than the effect of IgG1 and IgG3 (Figure 2C). Correlational analysis across all antibody functions demonstrated positive relationships between all antibody functions, highlighting the overall coordination of the functional humoral immune response across vaccinees (Figure 2D). Thus, despite the observed variation (Figure 2, A and B), all functions were associated with IgG1, IgG3, and — surprisingly — IgA levels (Figure 2E), highlighting the population level associations but clear individual variation. These data point to significant heterogeneity in polyclonal antibody functional programming, along with the unexpected correlation of both correlates of reduced and enhanced risk of infection in tuning antibody effector function.

### IgG1 is the major driver of effector functions, supported by IgG3 and IgA1.

**Figure 3:**
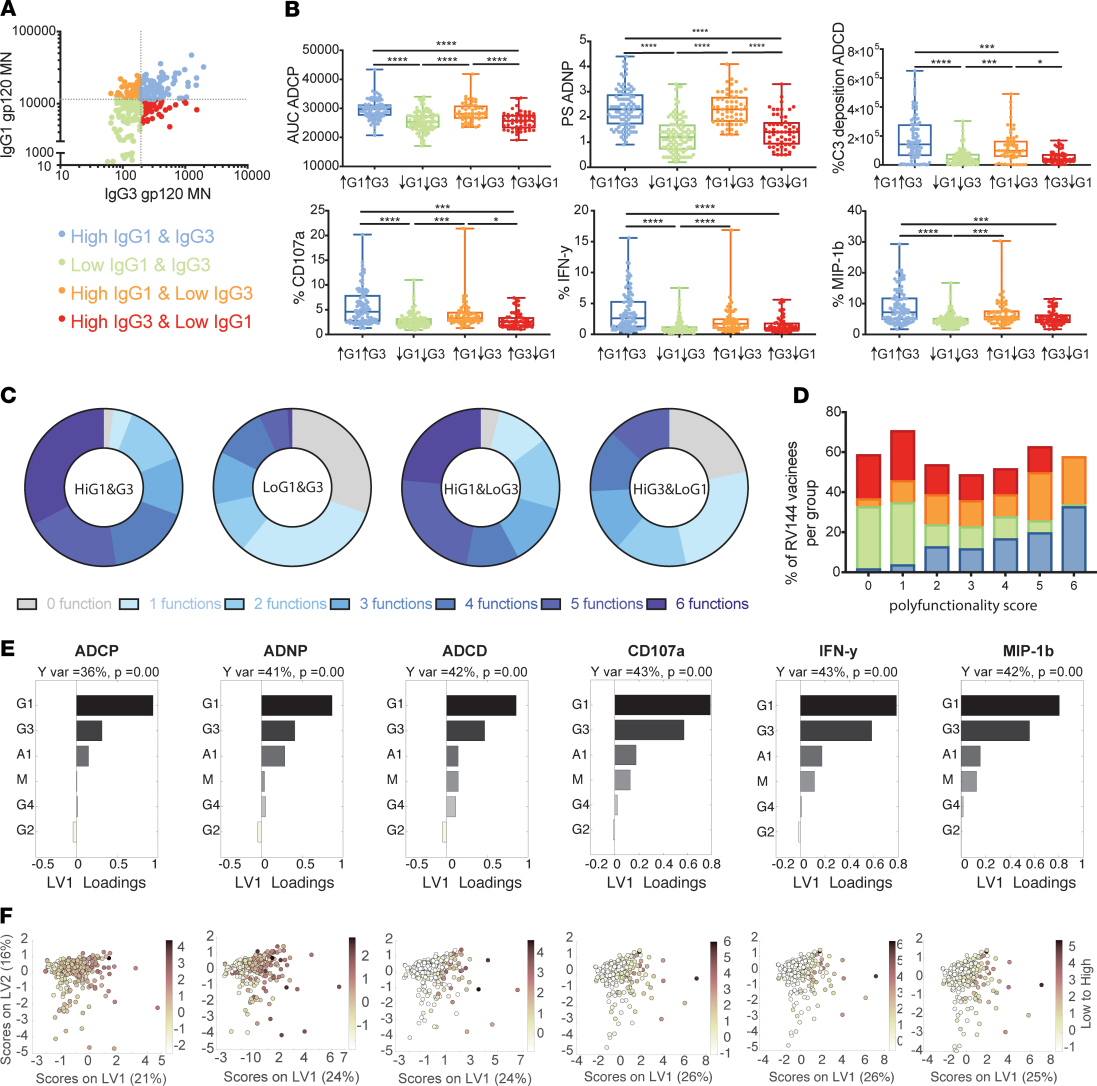
IgG1 is the major driver of effector functions in RV144 vaccinees. (**A**) The correlation plot depicts the relationship between IgG1 and IgG3 levels to gp120 MN across all 300 vaccinees. RV144 vaccinees were divided into 4 groups based on their IgG1 and IgG3 levels against boosting antigen gp120 MN above or below the median across all 300 vaccinees. (**B**) The whisker box plots individually depict the functional activity across the 4 IgG1/IgG3 groups. ADCP, ADNP, and ADCD levels are shown in the upper row. NK cell–related functions are shown in the lower row. A Kruskal-Wallis test was performed to assess statistical differences between groups. *P < 0.05, ***P < 0.001, ****P < 0.0001. (**C**) The donut plot depicts the polyfunctional profile across the 4 groups, calculated as the number of functions within the group above the overall 300 individual median for each function. (**D**) The stacked bar graph depicts the polyfunctionality distribution (0 functions, 1 function, 2 functions, etc.) across the 4 groups. (**E**) OPLSR analysis for each Fc-mediated effector function against antibody subclass/isotype levels. The bar graph shows the loadings on LV1, where the variable importance in projection (VIP) score is plotted for each subclass/isotype. The direction of the bar depicts if the isotype/subclass positively influences antibody Fc-effector function within the model. *P* indicates Wilcoxon permutation test. (**F**) The score plots are shown corresponding to the loading plots for the OPLSR models. Each scatter plot shows the scores for a different effector function, corresponding to the order in **E**.

To begin to define the combinatorial effect of antibody subclasses, the 300 vaccinees were split into 4 groups based on their IgG1 and IgG3 relationship against gp120 MN: IgG1^hi^/IgG3^hi^, IgG1^hi^/IgG3^lo^, IgG1^lo^/IgG3^hi^, IgG1^lo^/IgG3^lo^ (Figure 3A). Functional activity was then assessed across the 4 groups. Across all functions, subjects with high levels of both vaccine-specific IgG1 and IgG3 levels exhibited the highest functional activity. However, these responses were not significantly different than those elicited by individuals with high IgG1 in the absence of IgG3 responses, suggesting that — while IgG3 may correlate with functional activity — IgG3 was not required to elicit strong antibody effector functions. Moreover, individuals with IgG3 responses in the absence of high IgG1 elicited significantly lower levels of nearly all functions compared with individuals with higher levels of IgG1, arguing that IgG3 alone may be insufficient to drive high levels of antibody effector function. Therefore, while IgG3 was associated with enhanced antibody functionality (Figure 3B), IgG3 requires the presence of IgG1 to mediate robust functionality.

Previous analyses in spontaneous controllers of HIV pointed to a critical role for IgG3 in enhancing the polyfunctional profile of HIV-specific antibodies (17). Thus, we next examined the polyfunctional profile of HIV-specific antibodies across different IgG1/IgG3 vaccine groups. Despite the presence of similar univariate functional levels across all assays among individuals with high IgG1 levels, irrespective of IgG3 levels, vaccinees with higher IgG1 and IgG3 levels harbored a more polyfunctional antibody profile (Figure 3, C and D) compared with all other groups. These data illustrate the critical interaction between IgG1 and IgG3, where IgG3 maximizes antibody effector functional potential, driving enhanced polyfunctionality.

To gain a deeper appreciation for the contribution of IgG3 in driving functions second to IgG1, orthogonalized partial least squares regression (OPLSR) analysis was performed for all samples to define the importance of each subclass/isotype in driving antibody effector function; thus, isotype/subclass data were regressed onto each functional measurement separately. Similar to the correlation analysis (Figure 2D), IgG1 and IgG3 levels were among the top predictors of antibody effector functions. Specifically, IgG3 was more important in predicting NK cell–activating antibody functions compared with phagocytic functions (Figure 3E). Conversely, although IgA levels were correlated to antibody effector functions (Figure 2D), IgA levels were less important for predicting antibody effector function compared with IgG1 and IgG3 levels (Figure 3, E and F). However, because IgA responses were always positively associated with antibody effector function, these data strongly argue that IgA does not block antibody effector function in vitro, as previously suggested in monoclonal studies (7). Instead, these data suggest that, while IgG1, IgG3, and IgA were all induced in a coordinated manner at a polyclonal level, the levels of vaccine-induced IgG1 were most critical for driving effector functions, with variable contributions by IgG3, and a minimal but positive influence of IgA responses.

### Presence of IgA1 does not reduce functionality.

**Figure 4:**
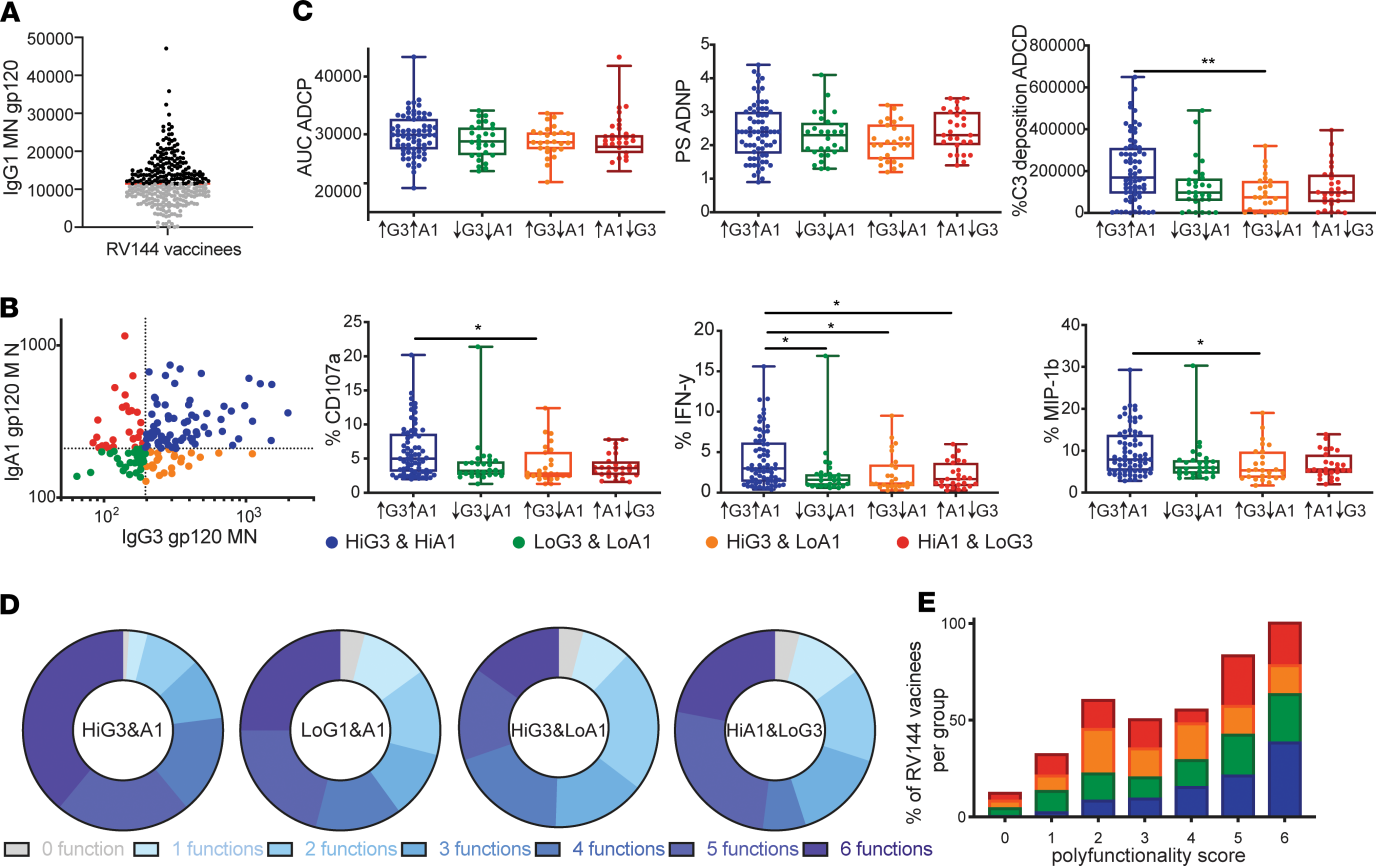
IgA levels are associated with greater antibody polyfunctionality. (**A**) The dot plot represents the overall IgG1 levels across all 300 RV144 vaccinees to the boosting antigen gp120 MN. (**B**) The correlation plot depicts the relationship between IgA1 and IgG3 responses across the top IgG1 responders. Subjects were then grouped based on median splits along both axes into high responses across both, either, or neither subclass/isotype. (**C**) Each graph shows the individual functional (ADCP, ADNP, and ADCD [top]; NK cell degranulation: CD107a%, IFN-γ%, and MIP-1β% [bottom]) across the 4 groups. A Kruskal-Wallis test was performed for statistical comparison between the groups. *P < 0.05, **P < 0.01. (**D**) The donut plots depict the polyfunctionality profiles across the 4 groups. (**E**) The stacked bar graph shows the overall distribution of functions across the groups.

Given that IgA was positively associated with antibody effector function within polyclonal antibody functional assays, these data seem to refute the previous notion that IgA may impede IgG1-mediated antibody effector functions (7). Thus, we aimed to probe the possibility that IgA may have other functional consequences (11). Substantial variation in IgA1 levels were observed across vaccinees (Figure 4A). Thus, 150 vaccinees with IgG1 levels against gp120 MN above the median were selected to specifically examine the potential antagonistic role of IgA on IgG1 function. These individuals were then split based on the correlates of reduced (high IgG3) or enhanced (high IgA) risk of infection and were divided into 4 groups based on IgG3 and IgA1 levels (Figure 4B). Limited differences were observed across all 4 groups with respect to ADCP and ADNP activity, highlighting the dominant role for IgG1 levels in this polyclonal pool, rather than IgG3 or IgA levels in shaping these responses. Conversely, individuals with high IgG3 and IgA levels exhibited enhanced ADCD and NK cell functions compared with individuals with high IgG3 and low IgA levels, arguing for a positive benefit for the presence of both classes in driving the most functional antibody responses (Figure 4C). Similar data analyses with IgA2 showed the same trend of IgA2 being associated with higher antibody functionality; however, IgA2 levels overall were too low to draw significant and relevant comparisons between groups.

These data point to the possibility that IgA levels may enhance all antibody effector functions, with a preferential induction of complement and NK cell activity. Thus, given that all antibody effector functions were correlated (Figure 2C), it is plausible that ADCD and specific NK cell functions may be a liability for the protective immune response, while ADCP and ADNP, which were not significantly affected by IgA levels in this polyclonal sample pool, may represent correlated and preferentially protective humoral immune functions. Moreover, polyfunctional profiling highlighted the highest level of polyfunctionality among the groups with high IgA levels (Figure 4, D and E) and a clear tendency toward higher numbers of functions among subjects with IgA responses; however, the differences in polyfunctionality were not significant via Kruskal-Wallis test. In light of IgA being a correlate of risk (13), these data suggest that polyfunctionality may not be the ultimate correlate of protection; instead, the induction of specific combination of functions, rather than all functions, may play a more important role in protection.

### IgA1 positively contributes to ADNP, ADCD, and CD107a degranulation but inhibits IFN-γ and MIP-1β cytokine secretion.

**Figure 5:**
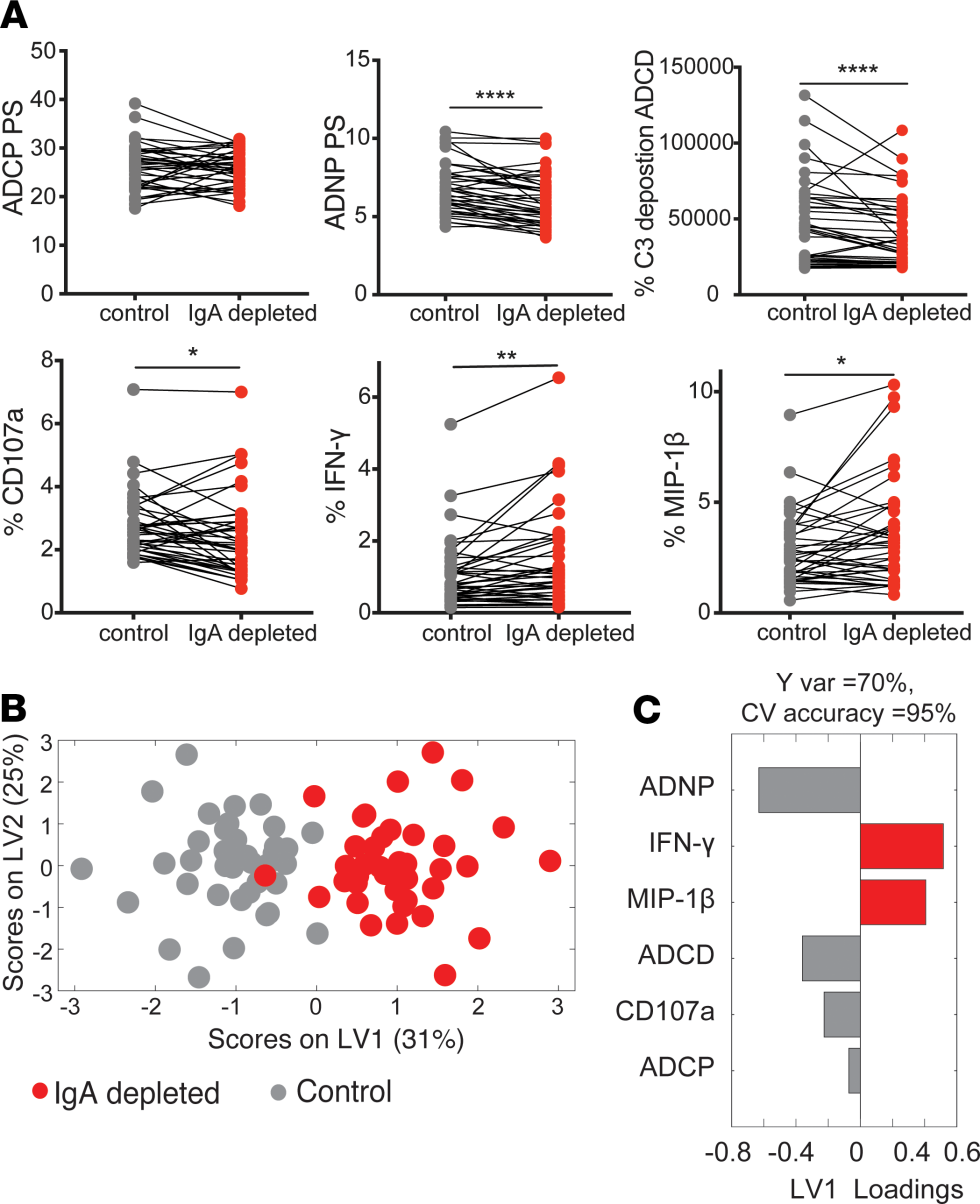
IgA both blocks and drives effector activity. (**A**) The line graphs depict the overall functional activity of 40 IgA-depleted and undepleted plasma pairs across all 6 effector functions against gp120 MN. Top panel shows ADCP, ADNP, and ADCD assays; the lower panel compares the sample groups for NK cell functions (CD107a, IFN-γ, and MIP-1β). Pairwise *t* tests were used to compare differences across groups. *P < 0.05, **P < 0.01, ****P < 0.0001. (**B**) An MLPLSDA was used to define the specific features that most effectively provided resolution between the antibody profiles of the control and IgA-depleted plasma pairs. Dots represent individual samples (control, gray; IgA depleted, red). The orthogonalized approach ensured that latent variable 1 (LV1) captured the separation between IgA-depleted and undepleted antibody profiles, while LV2 captured the antibody profile variances that do not contribute to this separation. Five-fold CV was performed, resulting in 95% CV accuracy. (**C**) The bar graph shows the loadings on LV1, ordered by their enrichment in either IgA-depleted (right/red) or control (left/grey) profiles. Features were ordered based on their variable importance in projection (VIP) scores.

The positive relationship between IgA and antibody functionality within polyclonal pools of antibodies raised the possibility that IgA may not block all functions but may selectively antagonize some functions or drive activity that may not provide protective activity. To therefore define the role of IgA, IgA was depleted from polyclonal antibody pools from 40 RV144 vaccinees, covering a spectrum of IgG and IgA titers. Depletion of IgA from plasma did not alter ADCP activity, suggesting that IgA does not block this particular activity (Figure 5A). Conversely, IgA depletion reduced ADNP, ADCD, and antibody-dependent NK cell degranulation, suggesting that IgA does not block these biological activities of IgG but rather contributes productively to driving these functions. While no effect of IgA on ADNP was detected in the previous analysis involving grouped samples (Figure 4), the depletion of IgA provided a means to capture the effect of IgA on neutrophil phagocytosis and showed that IgA is contributing to neutrophil phagocytosis. Surprisingly, removal of IgA resulted in increased levels of IFN-γ and MIP-1β secretion, providing support for the blocking activity of IgA for specific NK cell functions that may be essential for driving the recruitment and activation of an antiviral state for the destruction of infected cells.

These findings suggest that IgA may not block all antibody functions but may augment some functions while blocking others. To further explore the multivariate impact of IgA depletion and gain a sense for the functions most affected by IgA depletion, all functional data were collated, and the impact of IgA depletion was explored in multivariate space. IgA depletion led to a significant shift of the Fc-mediated, antigen-specific antibody profile of polyclonal sera (Figure 5B). Specifically, IgA depletion augmented 2 antibody functions, NK cell IFN-γ and MIP-1β secretion, highlighting the highly specialized negative impact of IgA responses in blocking these functions (Figure 5C). Conversely, the presence of IgA was associated with significant ADNP, ADCD, and antibody-dependent NK cell degranulation, showing that IgA contributed to the induction of these specific effector functions against the gp120 MN antigen. These data suggest both a blocking and functional role for IgA in polyclonal antibody effector activity, with IgA enhancing ADNP, ADCD, and antibody-dependent NK cell degranulation and selectively blocking NK cell cytokine secretion. While bulk IgA was depleted, functions were assessed against gp120 MN, ensuring that only the depletion of vaccine-specific IgA was relevant for the measurement of functional responses. Since IgA was associated directly with risk of infection in RV144 vaccinees, functions enhanced by this isotype might have a dispensable role in protective immunity; however, the importance of other IgA types such as secretory IgA in mucosal areas would need to be analyzed in future vaccine trials with available mucosal samples to comprehensively decipher the role of IgA.

### Case/control data confirm role of IgA in some functional responses.

**Figure 6:**
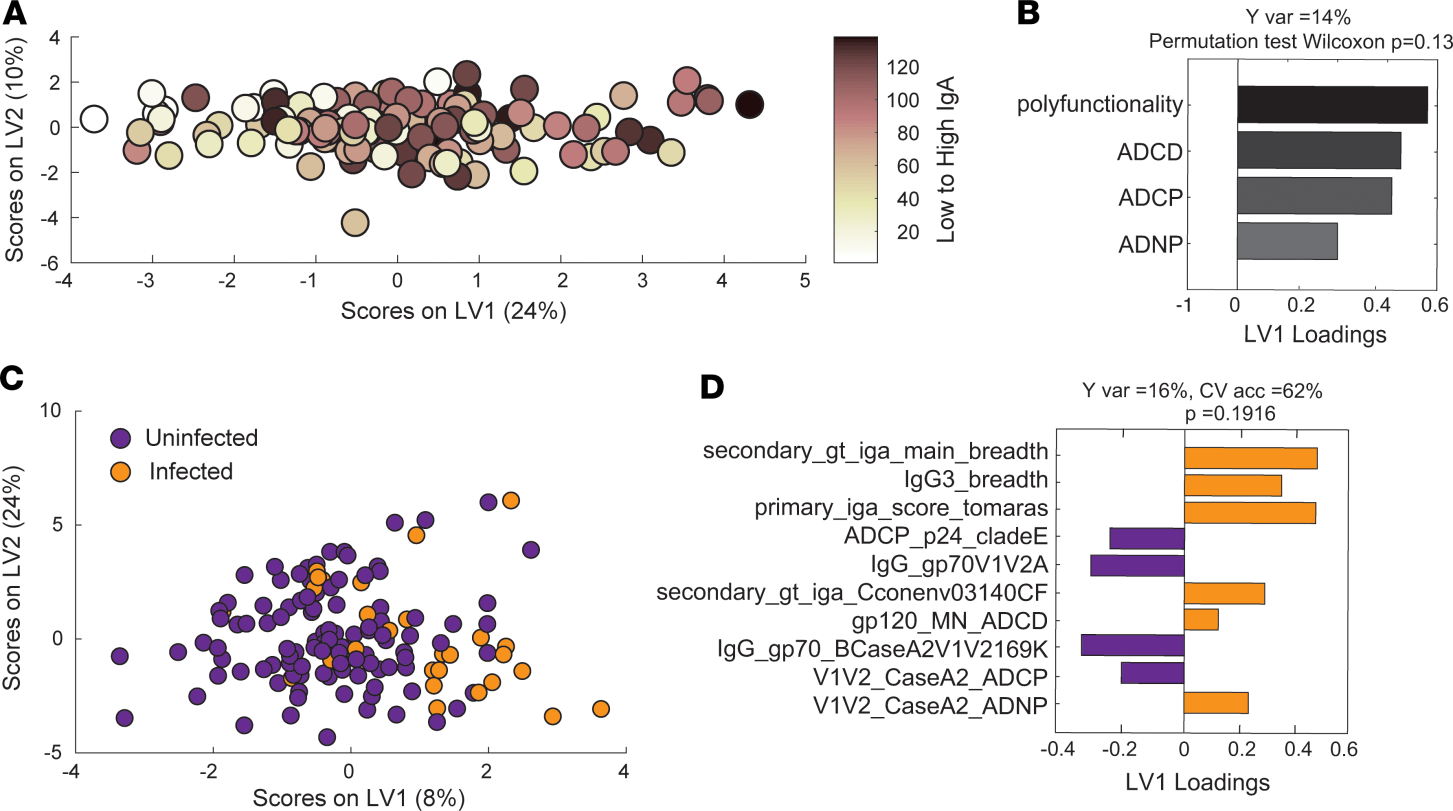
IgA is associated with function in case/control study. Functional and biophysical measurements from 110 vaccinated controls and 28 vaccinated cases from the RV144 vaccine trial were evaluated. (**A**) A PLSR model was developed to define the functions associated with increasing levels of IgA. Dots represent vaccinees and are colored according to their rank of low to high IgA score (dark, high; light, low). Features associated with increasing IgA are captured on LV1, accounting for 24% of the variance of IgA. To assess the model significance, permutation test was performed by randomly shuffling the labels. This model outperformed 87% of random models. (**B**) The bar graph shows the loading plot for LV1, in which all functions were ranked based on their VIP scores, which provides a measure of their importance in driving separation of the groups in association with IgA levels. Only loadings with VIP scores > 1 are shown. (**C**) The OPLSDA was used to analyze the features separating uninfected and infected vaccinees. LV1 and LV2 account for 8% and 24% of the variability in the measured antibody features, respectively. The separation of cases and controls was mostly captured on LV1, capturing 16% of the Y variation. Conversely, LV2 captures the variability in the antibody features that do not contribute to infection status. A 5-fold CV of the model resulted in a CV accuracy of 62%. Permutation test was performed by randomly shuffling the labels. The OPLSDA model performed better than 89% of random models (Wilcoxon, *P* = 0.19). (**D**) The bars in the loadings plot are ranked based on their VIP scores; orange features are enhanced in the vaccinated cases group, whereas purple variables are enriched in vaccinated controls.

In order to validate the apparent dual role of IgA in enhancing ADCD, ADNP, and NK cell–associated cytokine secretion, as well as hampering CD107a expression, we analyzed functional responses in case/control samples from the RV144 vaccine trial across uninfected (controls) and infected (cases) vaccinees. The case/control data set allowed for the investigation of the functional impact of IgA on shaping protective immunity. Using a Partial least squares regression (PLSR) analysis, we initially probed the impact of IgA on shaping antibody effector function across all vaccinees by regressing antibody profiles on IgA levels (Figure 6A). The loading plots illustrate the association between high IgA levels and polyfunctionality, as well as ADCD, ADCP, and ADNP (Figure 6B), as previously observed at the univariate level in unexposed vaccinees (Figure 4B and Figure 5). Thus, the PLSR analysis confirms the functional, rather than blocking, role of IgA in the case/control cohort.

To dissect the difference in effector functions between infected and uninfected vaccinees, an orthogonal partial least square discriminant analysis (OPLSDA) was performed to separate the 2 vaccinated groups based on physical and functional measurements (Figure 6C). While vaccine-induced antibody effector function was unable to completely separate out cases from controls, cases were clearly enriched on the right side of the dot plot. Importantly, since it is unclear whether the controls in this same region of the plot were truly exposed, it is plausible that this region represents the “risk” profile across all vaccinees.

Deeper analysis of the features that were selectively enriched in individuals on the right side of the plot revealed higher levels of IgA, higher IgA breadth, and — interestingly — higher levels of IgG3 breadth (Figure 6D). This counterintuitive relationship between the correlate of reduced and enhanced risk of infection exists due to the related but nonlinear relationship of these 2 antibody isotypes. Specifically, within a polyclonal antibody pool, the ratio of each isotype determines the overall functionality of vaccine-induced antibodies. Thus, at higher IgA levels, IgG3 activity may be overcome, and vice versa. Thus, these relationships point to a more complex interaction between the different isotypes and susceptibility.

Moreover, the right side of the plot also was enriched for enhanced gp120 ADCD and V1V2-specific ADNP, highlighting once again the potential role for complement and neutrophils as risk factors that track with IgA levels (Figure 6D). Conversely, on the left side of the plot, ADCP was clearly enriched in uninfected vaccinees. This function was associated with higher levels of IgG responses to V1V2 in the uninfected group, pointing to a critical role for IgG-induced phagocytosis as a possible critical correlate of reduced risk of infection.

## Discussion

Despite the disappointing results from the South African HVTN702 study (18), defining the unique humoral immune responses that emerged in the RV144 HIV-1 vaccine trial may provide clues to guide a future vaccine design against HIV. Several changes in the HVTN702 trial protocol — including changes in the antigen insert, addition of a late boost, and potentially novel adjuvants, as well as vaccination in populations with distinct immune backgrounds — likely all led to alterations in antibody profiles among HVTN702 and RV144 vaccinees. However, to begin to define the underlying mechanisms that may have led to differences in vaccine outcomes across the studies, here we aimed to define the landscape of humoral immune profiles, previously linked to risk of infection, across a large group of RV144 vaccinees. Given the univariate importance of IgG1, IgG3, and IgA responses, we initially profiled the relationship and interactions between these antibody classes to define the polyclonal synergistic and antagonistic relationships of these antibodies with respect to antibody effector function.

Heterogeneity in antibody levels was observed across antigens and antibody subclasses, highlighting the diversity of antibody profiles induced among RV144 vaccinees (Figure 1). Although antigen-specific responses correlated highly within subclasses and isotype, V1V2-specific response correlated with each other but not with gp120-specific responses and vice versa. These data point to epitope-specific heterogeneity in humoral immune profiles, with largely correlated IgG1 and IgG3 responses. Similarly, previous studies noted epitope-specific differences in subclass selection across V1V2- and gp120-specific responses across RV144 and VAX003 trials (8). While the studies performed here focused on the analysis of IgG1, IgG3, and IgA responses against recombinant monomeric gp120 MN, further delineation on trimeric or soluble envelopes may reveal additional epitope-specific changes. Moreover, recent analyses of the HVTN505 vaccine trial have also shown that IgG3 breadth correlated with reduced risk of infection (19), highlighting the importance of a broad IgG3 response against various epitopes. Moreover, here we noted that IgG1 and IgA responses were dominantly focused on the priming immunogen, whereas IgG3 responses were largely directed at the boosting antigens, suggesting a more recent selection of IgG3 by the vaccine regimen. Given that IgG3 is the first IgG subclass in the IgH locus (20), followed by IgG1 and then IgA1, these data potentially point to subclass selection biases driven by time since last vaccination. Thus, the selection of a more recent response (marked by IgG3) to the boosting antigens may drive enhanced protective immunity. Whether this is related to complete class switch of priming responses following boosting, compared with unswitched responses to the boost, is unclear. Moreover, aside from the choice and order of antigens in the vaccine, it is well documented that the choice of delivery strategy for the antigen is important. Different vectors, such as Ad5, have shown to be highly immunogenic and can be used both as prime or boost, whereas DNA for example is a well-characterized choice for a prime (21, 22). While this study allowed for the investigation of the humoral profile to different antigens included in prime and boost, it should be further decoded how prime/boost strategies will affect the IgG1/IgG3 distribution. However, this subclass/isotype-specific dominance pattern may point to a potential need to boost with the most critical epitopes at the final vaccine time, which points to a shift in the response to more protective subclasses (23).

Because both IgG1 and IgG3 were associated with reduced risk of infection (8, 16), we investigated the functional contribution of these subclasses in a polyfunctional sample pool. Elevated IgG1 and IgG3 levels were associated with more polyfunctional antibody profiles, recently associated with protection (9), and particularly with elevated NK cell activity (Figure 2), as previously observed (8, 17). The increased functionality in individuals with both high levels of IgG1 and IgG3 points toward a critical synergy between these subclasses, potentially via the synergistic collaboration of the subclasses within an immune complex upon Fc-receptor (FcR) binding on innate immune cells. However, despite these associations, limited univariate functional differences existed in the functional response among individuals with high IgG1 levels, irrespective of their IgG3 response, pointing to the ability of IgG1 to compensate for lower IgG3 levels. Similarly, IgG3 depletion in previous studies resulted in partial but incomplete depletion of antibody effector function (9). Thus, these data suggest that IgG1 antibodies were largely responsible for driving antibody effector functions, indicating that IgG3 is a biomarker but a potentially nonessential mechanistic correlate of immunity.

The correlation of vaccine-induced IgA responses to risk of HIV acquisition (12) was thought to be potentially attributable to the blockade of IgG1 function, especially of ADCC (7). Here, we observed both blocking as well as potentially deleterious IgA functions that may have collectively or individually contributed to differential risk of infection. Specifically, IgA levels were strongly associated with all antibody effector functions in a polyclonal pool of uninfected vaccinees (Figure 2D), as well as in a case/control study (Figure 6B), and enhanced polyfunctionality was observed in individuals with high IgG1/IgG3/IgA responses (Figure 4D). The depletion of IgA resulted in a selective loss of complement activity, neutrophil phagocytosis, and NK cell degranulation, suggesting that IgA contributed directly to driving these functions (Figure 5A). In contrast, IgA impeded NK cell cytokine and chemokine secretion, pointing to a disconnect in isotype/subclass-driven individual NK functions. Since bulk IgA and not only vaccine-specific IgA was depleted from polyclonal samples, it remains to be investigated if IgA in general or only gp120 MN–specific IgA depletion would have had an effect on functions. However, the here-performed functional assays only detected antigen-specific effects due to the antigen-coupled bead-based method; it is unlikely that free circulating antibodies would affect the measurements. Therefore, these depletion data suggest that, while vaccine-specific IgA antibodies compete with IgGs and block NK cell cytokine production (7), RV144-induced IgAs may enhance NK cell degranulation, which is linked to NK cell cytotoxicity (24), which is linked to reduced risk of infection (6). Given the importance of NK cell cytokines in polarizing innate immune function, it is possible that NK cells may promote an antiviral state, driving enhanced monocyte phagocytosis and reduced CD4^+^ T cell permissivity, rather than directly contributing to cytotoxic destruction of infected cells.

Using case/control data, IgA tracked with antibody effector function, and most tightly with ADNP and ADCD responses, suggesting that rather than blocking these functions, IgA may synergize with IgG to drive this activity (Figure 6, C and D). However, whether these functions are protective or hinder protection is unclear. The complement system plays a controversial role in the context of HIV vaccine–mediated protection and infection, as a complement has been shown to drive viral lysis, but the virus also exploits complement regulators to escape elimination (25). Likewise, neutrophil phagocytosis has been implicated in protection in mucosally vaccinated nonhuman primates (26), but neutrophils also degranulate and secrete copious inflammatory modulators that have been implicated in susceptibility to HIV infection within mucosal tissues (27, 28). However, here we investigated plasma IgA due to the lack of mucosal samples from the RV144 cohort, in order to address the importance and relevance of IgA and the connection to functionality; in the future, IgA in mucosal secretions should be analyzed when mucosal sampling is available in other trials. ADCP levels were higher in uninfected vaccinees in the case/control analysis and were unlinked from IgA levels, pointing toward a potentially protective role for this specific Fc-effector function, previously linked to protection in several nonhuman primate challenge studies (26, 29). Thus, ADCP in conjunction with NK cell cytokine secretion, which was selectively blocked by IgA in RV144 vaccinees, may collaborate to confer protection. These data potentially suggest that the presence of antibodies capable of eliciting ADCP and NK cell antiviral signals, rather than all antibody effector functions collectively, may be linked to reduced risk of infection.

The data presented here demonstrated that the RV144 vaccine elicited a highly heterogeneous, polyfunctional humoral immune response marked by variable coelicitation of IgG1, IgG3, and IgA responses to the vaccine. The combinatorial elicitation, rather than the discrete induction of any one of these subclasses/isotypes, was associated with distinct functional antibody profiles, pointing to different patterns of effector functions that may be associated with differential levels or protection or susceptibility. Ultimately, the data show the synergistic function of IgG3 and IgG1 in driving enhanced antibody effector profiles, and the functional as well as blocking role of IgA in tuning innate immune effector functions. The data presented here suggest that, to achieve maximal clearance and control of HIV, cooperativity between antibody-driven NK cell cytokine secretion and ADCP are required. However, with the changes to the vaccine regimen/population in HVTN702, it will now be important to determine whether these RV144 multivariate correlates were simply not induced in HVTN702 vaccinees or whether the force of infection in South Africa may have overwhelmed these antiviral humoral immune functions.

## Methods

### Samples.

Initial results of this trial were first published by Rerks-Ngarm et al. (3). Patients enrolled in the RV144 trial were vaccinated with an ALVAC-HIV canarypox vector (vCP1521) at weeks 0 and 4, and a combination of the ALVAC-HIV vector and the recombinant protein immunogen AIDSVAX B/E (a heterologous gp120 protein regimen including 2 gp120 antigens derived from MN and A244) was administered at week 12 and 24 (ClinicalTrials.gov; NCT00223080). The 300 plasma samples analyzed in this study were obtained 2 weeks after the last boost, at the peak immunogenicity time point at week 26 (3). In addition, data from the original case/control study, of 110 uninfected and 28 infected RV144 vaccinees, were included (6). All individuals completed the whole vaccination schedule of RV144.

### ADCP.

A bead-based assay was used to assess the ADCP by monocytes. The THP-1 based phagocytosis assay was performed as previously described (30). Briefly, avi-tagged biotinylated (Avidity, BirA500) gp120 MN used in the RV144 boost (Duke University) antigen was coupled to 1 μm yellow fluorescent neutravidin beads (Thermo Fisher Scientific) for 2 hours at 37°C. After removal of excess antigen by washing with 0.1% Bovine serum albumin (BSA) in phosphate buffered saline (PBS) for blocking, saturated beads (1.82 × 10^8^ beads/well) were incubated with the appropriately diluted sample (3 dilutions at 1:100, 1:500, and 1:2500) in order to calculate the AUC. Immune complexes were washed, and 2.5 × 10^4^ THP-1 cells (American Type Culture Collection) were added per well and incubated for 16 hours at 37°C. Cells were fixed in 4% PFA, and sample acquisition was performed via flow cytometry (Stratedigm, S1000EXi). Events were gated on single cells and bead-positive cells; a phagocytosis score was calculated by the percentage of bead-positive cells GMFI/10,000. The AUC was calculated using GraphPad Prism based on 3 dilutions and a duplicate.

### ADNP.

The ADNP assay was performed as described previously (31). In brief, neutravidin beads were saturated with biotinylated (Avidity, BirA500) antigen gp120 MN and incubated with 1:100 diluted plasma. Neutrophils were isolated from whole blood from healthy donors with Ammonium-Chloride-Potassium (ACK) lysis buffer (Thermo Fisher Scientific) via lysis of RBCs. Cells were washed, and 5 × 10^4^ cells were added per well and incubated for 1 hour at 37°C. Then, cells were stained with anti-Cd66b Pacific blue antibody (clone G10F5, catalog 305102, BioLegend) to identify CD66b^+^ neutrophils. Fixed cells were acquired via flow cytometry (Stratedigm, S1000EXi). The neutrophil population was defined as CD66b^+^, and a phagocytosis score was calculated as described above. Data presented are the average of 2 different blood donors.

### ADCD.

As described recently (32), biotinylated antigen gp120 MN was coupled to red fluorescent neutravidin beads (Thermo Fisher Scientific). Immune complexes were formed with plasma samples for 30 minutes at 37°C at 3 dilutions (1:100, 1:500, 1:2500) in order to calculate the AUC. Lyophilized guinea pig complement was resuspended according to manufacturer’s instructions (Cedarlane), and 2 μL per well was added in veronal buffer with 0.1% gelatin (Boston BioProducts). After 20 minutes of incubation at 37°C, immune complexes were washed and C3 was detected with a fluorescein-conjugated goat IgG fraction to guinea pig complement C3 (catalog 855385, MpBio). Complement-coated beads were acquired on a BD LSR II with a high-throughput sampler, and C3 deposition was reported by gating on single beads and C3^+^ events of 2 independent runs. AUC was calculated in Prism.

### NK degranulation.

An ELISA-based antibody-dependent NK cell activation assay was used to analyze NK activation and degranulation. ELISA plates (Thermo Fisher Scientific NUNC MaxiSorp flat bottom) were coated with gp120 MN (300 ng per well) at 37°C for 2 hours; plates were blocked with 5% BSA in PBS overnight at 4°C. A total of 50 μL of samples was added at a 1:50 dilution to each well and incubated at 37°C for 2 hours. Healthy donor buffy coats were used to isolate NK cells with RosetteSep (Stemcell Technologies), and NK cells were rested overnight, supplemented with 1 ng/mL of IL-15 (Stemcell Technologies). A total of 5 × 10^4^ NK cells with anti–CD107a-PE-Cy5 stain (clone H4A3, catalog 555802, BD Biosciences), brefeldin A (5 mg/mL) (MilliporeSigma), and GolgiStop (BD Biosciences) were added to each well and incubated for 5 hours at 37°C. NK cells were fixed and permeabilized using Perm A and B solutions (Thermo Fisher Scientific). Cells were subsequently stained for surface markers with anti–CD16 APC-Cy7 (clone 3G8, catalog 557758, BD Biosciences), anti–CD56 PE-Cy7 (clone B159, catalog 557747, BD Biosciences), and anti–CD3 Alexa Fluor 700 (clone UCHT1, catalog 557943, BD Biosciences). Intracellular staining included anti–IFN-γ APC (clone B27 [RUO], catalog 554702, BD Biosciences) and anti–MIP-1β PE (clone D21-1351, catalog 550078, BD Biosciences). Acquisition occurred by flow cytometry (BD LSR II) with a high-throughput sampler. NK cells were defined as CD3^–^, CD16^+^, and CD56^+^. The antibody-dependent NK cell degranulation assay was performed in duplicate across 2 blood donors.

### Subclassing and isotyping via Luminex.

A customized Luminex subclassing assay was used to quantify the relative concentration of antigen-specific antibody subclass and isotype levels, as well as Fcγ-receptor (FcγR) binding (33). Carboxyl-modified microspheres (Luminex) were coupled with different HIV antigens (Table 1; Duke Protein Production facility or Immune Technology Corp.). Coupling was performed by covalent N-hydroxysuccinimide–ester (NHS-ester) linkages via EDC (Thermo Fisher Scientific) and Sulfo-NHS (Thermo Fisher Scientific) according to the manufacturer’s instructions. a total of 1.2 × 10^3^ beads per Luminex region per well was used in Luminex assay buffer containing 0.1% BSA and 0.05% Tween-20 and was added to each well of a 384-well plate (Greiner Bio-one). Diluted plasma samples (FcγR binding and subclass/isotyping, 1:100) were added and incubated for 16 hours at 4°C and were rocked at 900 rpm. The microspheres were washed 3 times with 60 μL of Luminex assay buffer with an automated plate washer (Tecan). PE-coupled IgG1-, IgG2-, IgG3-, IgG4, IgA1-, IgA2-, IgM-, or bulk IgG–specific detection reagents (Southern Biotech) were added at 1.3 μg/mL and incubated with immune complexes for 1 hour at room temperature while shaking at 900 rpm. The coated beads were then washed and read on a Bio-Plex 3D System. Similarly, for the FcR binding profiles, recombinant FcγRIIA, FcγRIIB, FcγRIIIA, and FcγRIIIB (Duke Protein Production facility) were biotinylated (Thermo Fisher Scientific) and conjugated to Streptavidin- PE for 10 minutes (Southern Biotech). Samples were run in duplicate per each secondary detection agent.

### IgA depletion.

For depletion of IgA from plasma samples, an affinity matrix of agarose-beads against IgA (Thermo Fisher Scientific) was used. The resin was coated with a single domain fragment specific to human IgA (Thermo Fisher Scientific). Following the manufacturer’s instructions, spin columns (Thermo Fisher Scientific) were packed with 50 μL of resin, and diluted plasma was added and incubated for 1 hour at room temperature. Columns were spun, and depleted plasma was recovered. Control plasma was incubated in resin without IgA-depleting antibodies to assess the nonspecific binding and run side by side in all assays as a control. Assays were run in duplicate with control and IgA-depleted plasma.

### OPLSR and OPLSDA.

To mathematically identify the antibody isotypes and subclasses contributing to the variation in Fc-mediated effector functions in Figure 3, E and F, and to rank the association of IgA score with functions in a case/control study in Figure 6, A and B, an OPLSR modeling framework was used (34). PLSR is a multivariate regression technique where linear combinations of features are used to predict the variance in the dependent variables. The model is then orthogonalized such that Latent Variable 1 (LV1) captures the variance in features that are in the direction of the dependent variable, while other latent variables describe the variation orthogonal to the predictive component. For the OPLSR model, variables were centered and scaled to an SD of 1. Five-fold cross-validation (CV) was performed on the data (Venetian blinds). To assess model significance, a permutation test was performed on cross-validated models by randomly shuffling the labels.

For the regression of antibody subclass and isotype contribution on each individual effector function in Figure 3, E and F, the variables IgG1, IgG2, IgG3, IgG4, IgA1, and IgM were used as input. IgA2 was intentionally excluded from this analysis, since the measured values were close to the PBS control levels. The model performed significantly better than random, with CV Wilcoxon *P* values of lower than 4 × 10^–6^ across functions. For regression on contribution of IgA score on functionality in the case/control set in Figure 6, A and B, input variables were functional measurements against gp120 MN (polyfunctionality, ADCD, ADCP, ADNP), V1V2 CaseA2 (ADCP, ADNP, ADCC, polyfunctionality), gp120 A244 (ADCP, ADCC), V1V2 A244 (ADCP), and p24 Clade E (ADCP). The model performed with a CV Wilcoxon *P* values of 0.13.

To identify the key features separating the 2 groups of infected and uninfected vaccinees in the case/control study in Figure 6, C and D, an OPLSDA was performed. While PCA is an unsupervised tool that simply examines overall variation in a data set, a PLSDA is a supervised method that aims to define discriminant features between groups. Coupled with feature downselection, PLSDA/PLSR provides a unique means to define features that directionally help resolve groups in a manner that avoids overfitting and overinterpretation of the data. This strategy makes the analysis and interpretation more accurate and provides tractable changes that may underlie vaccine group differences. The model used 26 variables, including functional and titer measurements; variables were centered and scaled to an SD of 1. Five-fold CV was performed on the data (Venetian blinds), resulting in a CV accuracy of 62% and a Wilcoxon *P* value of 0.1916. Loadings were ordered by their variable importance in projection (VIP) score, and loadings with a VIP score > 1 are depicted.

### Multilevel partial least squares discriminant analysis (MLPLSDA).

To determine the key features contributing to the profile differences between IgA-depleted and control plasma in Figure 5, B and C, an MLPLSDA framework was used (35). MLPLSDA uses the same principles as OPLSDA for multivariate data but also takes advantage of the paired structure of the data (plasma samples and their IgA-depleted counterparts). Intuitively, this analysis subtracts the effect of heterogeneity between IgA depleted/control pairs (inter-pair variability) and focuses on the effects within IgA/control samples. The model was constructed using the Fc-mediated functions as variables. These variables were centered and scaled to an SD of 1. Five-fold CV was performed on the data (Venetian blinds), obtaining a CV accuracy of 92%. LV1, which captured 21% of the variance in the antibody profile input (X) and 70% variance in the binary output Y, is in the direction of the separation of IgA-depleted and undepleted antibody profiles. To assess model significance, a permutation test was performed by randomly shuffling labels. The MLPLSDA model performed significantly better than random (Wilcoxon, *P* = 6 × 10^–6^).

### Statistics.

Data were analyzed using GraphPad Prism Version7 for Mac (GraphPad Software) for statistical significance and graphical representation. AUC was assessed for 3 sample dilutions of ADCP and ADCD using Prism. Polyfunctionality was calculated as the number of functions within the group above the overall 300-individual median for each function, with the maximum of polyfunctionality being 6. Correlations were calculated as Spearman’s correlations with a significant *P* < 0.05; *P* values in Figure 1C and Figure 2D were Bonferroni corrected for multiple comparisons. For analysis of 4 groups, Kruskal-Wallis test with Dunn’s multiple comparisons test was used; for comparison of IgA depletion data (2 groups), Mann-Whitney *U* tests were performed, with a significant *P* < 0.05. *P < 0.05, **P < 0.01, ***P < 0.001, ****P < 0.0001 for all analyses.

### Study approval.

Written informed consent was obtained from all volunteers, who were required to pass a written test of understanding (3), and this research was approved by the IRB of Massachusetts General Hospital, IRB approval no. 2015P000095. The investigators have adhered to the policies for protection of human subjects as prescribed in AR 70–25.

## Author contributions

GA designed the study. SF performed the immunologic assays; additional FcR binding data were received by MEA. SF, SD, and MFJ performed all analyses. SV provided study samples. SRN, PP, SN, and NM participated in study design and interpretation of data. MEA, HS, and and MFJ gave valuable comments and input on the manuscript. SF and GA wrote the paper with all coauthors. All authors contributed to the final version of the manuscript.

## Supplementary Material

supplemental data

## Figures and Tables

**Table 1:**
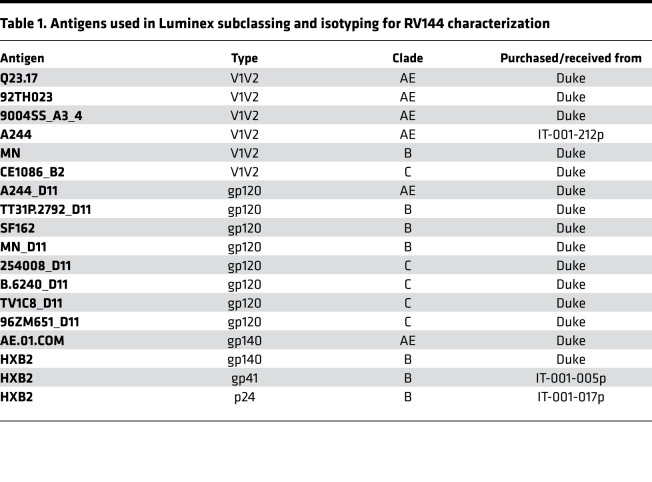
Antigens used in Luminex subclassing and isotyping for RV144 characterization

## Version 1 10/08/2020

In-Press Preview

## Version 2 11/05/2020

Electronic publication
