# Supplementary material for: IgG3 collaborates with IgG1 and IgA to recruit effector function in RV144 vaccinees
Source: JCI Insight. 2020 Nov 5;5(21):e140925. doi: 10.1172/jci.insight.140925 (PMC7710302; doi:10.1172/jci.insight.140925)
Supplement: supplemental data [file jciinsight-5-140925-s149.pdf]

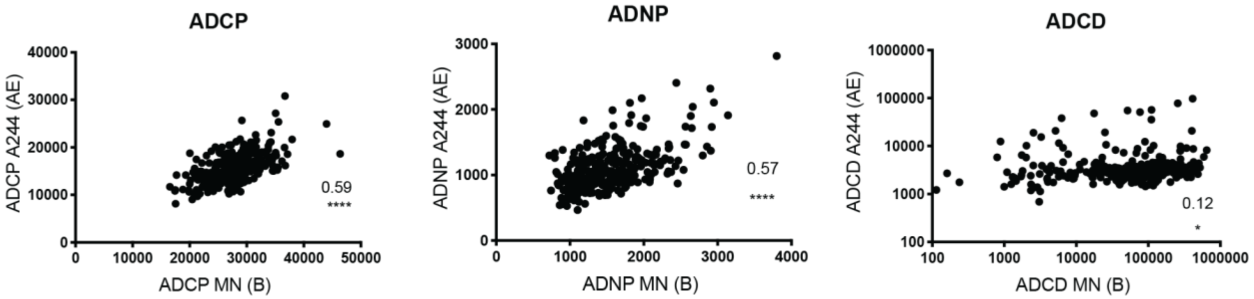

Supplemental Figure 1: Correlation between gp120 MN and A244 in effector functions. The correlation plots show Spearman correlations for ADCP, ADNP and ADCD between gp120 MN (Clade B) and gp120 A244 (Clade AE) antigens. The Spearman r value is indicated as well as the p value, **p<0.05, ***p<0.001, ****p<0.0001.*
